# Prevention of Fentanyl Induced Cough by Ketorolac: A Prospective, Randomized, and Double-Blind Study

**DOI:** 10.5812/aapm-161218

**Published:** 2025-07-19

**Authors:** Nasrin Nouri, Alireza Pournajafian, Sara Tahzibi, Soudabeh Djalali Motlagh

**Affiliations:** 1 Department of Anesthesiology, Firoozgar Hospital, School of Medicine, Iran University of Medical Sciences, Tehran, Iran; 2 Student Research Committee, School of Medicine, Iran University of Medical Sciences, Tehran, Iran

**Keywords:** Fentanyl, Cough, Lidocaine, Ketorolac

## Abstract

**Background:**

Fentanyl is often used during general anesthesia and can cause reflex cough.

**Objectives:**

We assessed the effectiveness of administering intravenous ketorolac in reducing the incidence and severity of cough caused by fentanyl, aiming to find an alternative to lidocaine in cases where it is contraindicated.

**Methods:**

In a prospective randomized controlled trial, a total of 210 patients, classified as American Society of Anesthesiologists (ASA) I or II, undergoing elective surgery, were randomly allocated to three groups: The Ketorolac group (K), the Lidocaine group (L), and the normal saline group (N). Three minutes before the injection of 3 mcg/kg intravenous fentanyl, patients received 0.5 mg/kg ketorolac, 1 mg/kg lidocaine, and 2 cc of 0.9% normal saline, respectively. The onset and severity of cough were documented within 3 minutes after the administration of fentanyl.

**Results:**

There was a significant difference in the onset time of cough between the groups (P = 0.001). Three individuals (4.3%) in group L and nine individuals (12.9%) in group N experienced severe coughs, with a significant difference in cough intensity among the groups (P = 0.001). There was no significant difference in heart rate (HR) and arterial oxygen saturation (SpO_2_) at different time points (P > 0.05) among the three groups. However, there was a significant difference among the groups regarding arterial blood pressure (BP) 3 minutes after fentanyl injection and 1 minute after intubation (P = 0.003, 0.001, respectively), with the mean arterial BP being higher in group N than in group L, and higher in group L than in group K.

**Conclusions:**

The administration of ketorolac and lidocaine decreases both the frequency and severity of cough induced by fentanyl, especially in individuals who received ketorolac, as no cases of severe cough were observed. Therefore, ketorolac can be used in cases where lidocaine is contraindicated to prevent coughing due to fentanyl.

## 1. Background

Fentanyl, a synthetic opioid, is commonly used for inducing general anesthesia to alleviate pain and anxiety related to surgical procedures (1-3). Paradoxically, it can cause coughing (4), which is usually transient, benign, and resolves on its own; however, it can occasionally be severe, spasmodic, and life-threatening (5). The resulting cough can increase intracerebral pressure, intraocular pressure, and intra-abdominal pressure, which may not be problematic in patients classified as American Society of Anesthesiologists (ASA) I or II. However, in patients who require a smooth induction of anesthesia, including those with high intracranial pressure, brain aneurysm, open eye injury, acute glaucoma, abdominal aortic aneurysm, pneumothorax, reactive airway, and full stomach, it may become problematic (6-8). Fentanyl-induced cough (FIC) can lead to cardiac arrhythmias, increased blood pressure (BP), airway obstruction, aspiration pneumonia, and other unwanted events that require immediate treatment during anesthesia induction (9-11). The incidence of FIC has been noted to be as high as 13% to 65%, and sometimes up to 80%, even with low doses of fentanyl (12-14). The mechanism of FIC is not completely understood, although several researchers have proposed different mechanisms; stimulation of the vagus nerve C-fibers on the proximal bronchus and inducing bronchoconstriction, activation of µ-opioid receptors in the prejunctional area, and release of histamine by lung mast cells may also play a role in this cough (15-19). Multiple predisposing factors for FIC have been identified, and several preventive measures have been proposed. Factors such as age, gender, weight, smoking, and opioid use (type, dose, concentration, injection rate, and injection site) are associated with FIC. It has been shown that increasing age and smoking, especially in females, are associated with an increase in the level and severity of FIC (20-24). Findings about the impact of the concentration and rate of fentanyl administration on FIC are controversial. Yeh et al.'s study showed that the average time for FIC to start after peripheral vein injection is 15 seconds, and if the injection is done within 30 seconds, a cough does not occur because its plasma concentration does not reach the necessary threshold for a cough response (25). Yu et al.'s study proved that diluting fentanyl with normal saline to 10 microg/mL obviates cough occurrence (26). Lin et al. showed that a longer injection time decreases the occurrence of cough induced by fentanyl (27). Various pharmacological and non-pharmacological methods have been reported for managing FIC before the induction of general anesthesia (1, 4, 15, 28, 29). The potential side effects, delayed onset or long duration of action, and limited effectiveness of some drugs like ephedrine, propofol, dexmedetomidine, magnesium sulfate, and lidocaine restrict their use in patients. Lidocaine is effective in reducing FIC, but high doses increase the risk of cardiovascular and neurologic side effects, including arrhythmogenic potential, vasodilatory effects, hypotension, tremor, and seizures. These risks are particularly relevant in high-risk patients, such as those with cardiovascular instability or a predisposition to arrhythmias (11, 13, 30, 31).

Evidence shows that non-steroidal anti-inflammatory drugs like aspirin (500 milligrams per day) and ketorolac tromethamine (0.5 mg/kg) significantly reduce cough. The antitussive mechanism of ketorolac may be associated with a reduction in histamine release (32, 33). Adverse effects of ketorolac are generally mild and include somnolence, dizziness, and gastrointestinal discomfort or bleeding, though these are rare with a single perioperative dose. Unlike lidocaine, ketorolac does not carry a risk of arrhythmia or significant cardiovascular depression, which may be advantageous in high-risk populations.

## 2. Objectives

This study aims to evaluate how effective ketorolac is in managing the cough reflex induced by intravenous fentanyl administration before general anesthesia, and to compare its effectiveness with intravenous lidocaine to find a less harmful alternative in high-risk patients.

## 3. Methods

This study was conducted as a prospective, randomized, double-blinded controlled trial at Hazrat Rasoul Akram Hospital, affiliated with Iran University of Medical Sciences, between July 2022 and March 2023. Ethical approval for this study was obtained from the Ethics Committee of Iran University of Medical Sciences (IR.IUMS.FMD.REC.1400.538) and was registered in the Iranian Registry of Clinical Trials with registry number IRCT20220102053599N1. Written informed consent was obtained from each patient, and the study protocol was conducted following the principles of the Helsinki Declaration.

A total of 234 patients with an ASA physical status of 1 or 2, aged 18 - 60 years, scheduled for elective surgery requiring general anesthesia, were included in the study. All patients with pulmonary emphysema, bronchial asthma, a history of upper respiratory infection in the last 2 weeks, smoking, a history of hypertension and coronary artery disease, chronic cough that made it difficult to distinguish FIC, high intraocular, intracranial, or intracranial pressure, taking anti-anxiety and anti-depressants before surgery, taking antitussives such as codeine, dextromethorphan, baclofen, steroids, or bronchodilators during the last week, BMI above 28 kg/m², history of chronic opioid use, taking cough medicines such as angiotensin-converting enzyme inhibitors and angiotensin receptor inhibitors, history of GERD or peptic ulcer, history of kidney or liver disease, pregnancy or breastfeeding, known hypersensitivity to fentanyl or ketorolac, G6PD enzyme deficiency disease, high risk of gastrointestinal bleeding, and unwillingness to participate in the study, were not included in the study.

All patients fasted for at least 6 hours after a light meal. None of the patients received any preoperative medication. Upon entering the operating room, electrocardiogram, non-invasive BP, heart rate (HR), and oxygen saturation (SpO_2_) were monitored, and the patients were allowed to lie quietly on the bed for about one minute. None of the patients received premedication. A 20-gauge angiocath was used to establish a peripheral vein on the back of each patient's non-dominant hand, and a three-way connector was attached to it. Ringer's lactate solution was started at a rate of 4 cc/kg/hr through the three-way connector. The height of the IV bag was about one meter from the patient. All patients received 3 L/min oxygen through a face mask for 5 minutes.

Patients received either 0.5 mg/kg ketorolac (group K), 1 mg/kg lidocaine (group L), or saline 0.9% (group N), in a syringe with a similar volume, injected through the three-way connector over 15 seconds, 3 minutes before the administration of fentanyl. After 3 minutes, all patients received 3 mcg/kg intravenous fentanyl within 3 seconds. Then, another anesthesiologist, unaware of the group assignments, recorded the onset and intensity of coughing within three minutes after the fentanyl injection. The patients were also kept unaware of the pretreatment medication. Cough severity was graded based on frequency: Mild (1 - 2), moderate (3 - 4), severe (5 or more) (12, 13). The mean arterial pressure (MAP), HR, and SpO_2_ were recorded before the administration of ketorolac, lidocaine, and normal saline (T0), 3 minutes after the administration of fentanyl (T1), and 1 minute after tracheal intubation (T2). If SpO_2_ fell below 90% while coughing, assisted ventilation was immediately started. General anesthesia with a similar protocol was administered to all patients after coughing cessation (if it occurred) or 90 seconds after fentanyl injection, consisting of propofol 2 mg/kg and cisatracurium 0.2 mg/kg. After manual ventilation for 5 minutes, intubation and mechanical ventilation were performed.

The primary endpoint is the occurrence of a cough within 90 seconds of fentanyl administration. The secondary endpoints are the onset time of cough, the number and severity of coughs during the 90 seconds after the administration of fentanyl, and changes in BP and HR. Data were analyzed using SPSS v.24 software. Quantitative data were displayed as mean ± standard deviation (SD), and qualitative variables as percentages. Demographic characteristics of patients were analyzed with the Student's *t*-test for continuous variables and the chi-square test for discrete variables. The prevalence of cough was compared using the chi-square test, and the severity of cough was analyzed with the Mann-Whitney U test between the three groups. Two-way ANOVA analysis was performed for changes in BP, HR, and SpO_2_ before and after fentanyl injection. The frequency of side effects, if any, was analyzed with the chi-square or Fisher's exact test. A P-value less than 0.05 was considered statistically significant.

## 4. Results

**Figure 1.**
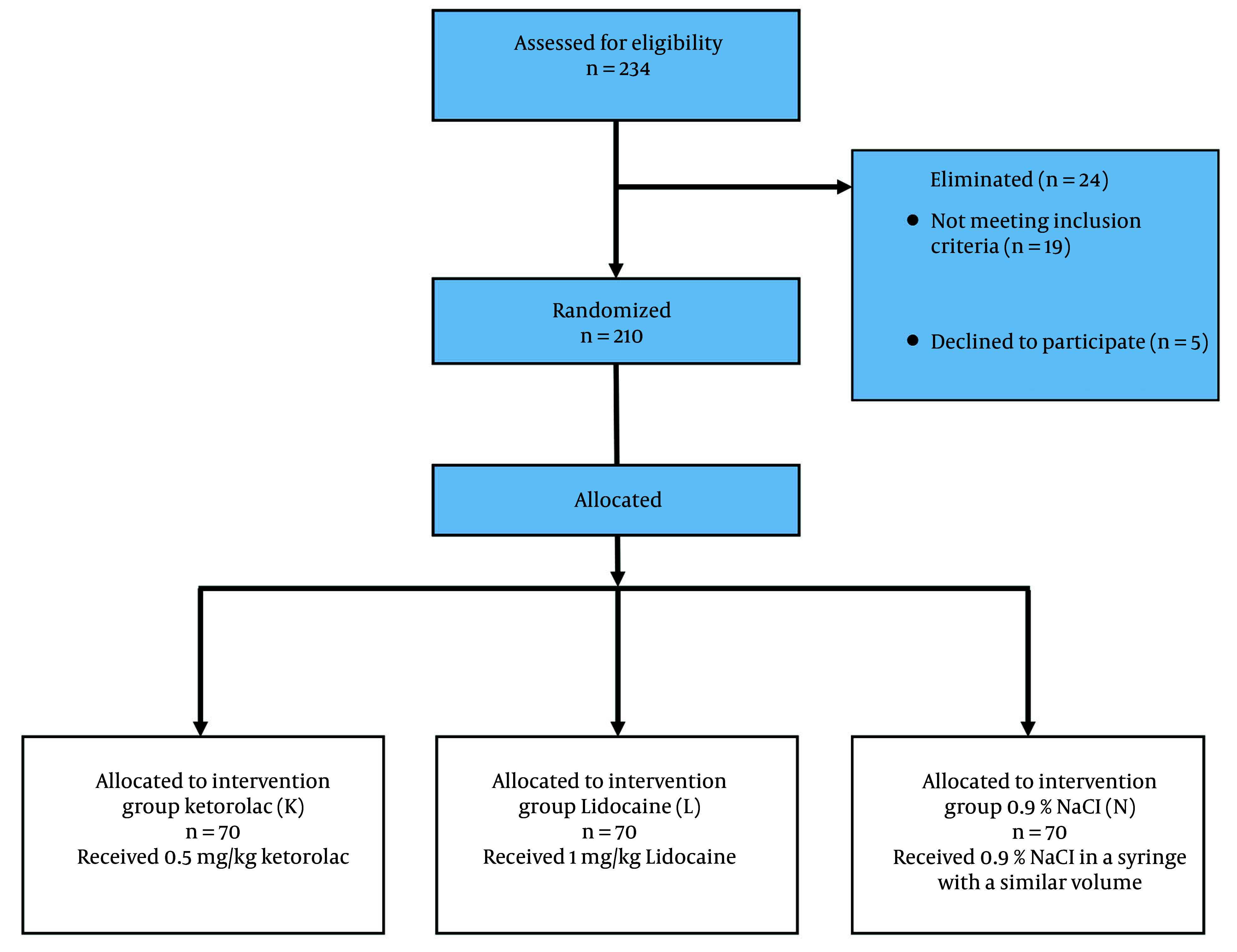
Consolidated Standards of Reporting Trials (CONSORT) diagram

In this study, two hundred and thirty-four patients were assessed for eligibility, 210 were included in the study, and all of the 210 participants completed the study. The study is presented in a Consolidated Standards of Reporting Trials (CONSORT) flow diagram (Figure 1). 

No statistically significant difference was observed between the groups in terms of age, sex, weight, or height among the 210 patients evaluated in the context of the study (P > 0.05) (Table 1). 

**Table 1.** Demographic Data ^a^

Variables	K Group (70)	L Group (70)	N Group (70)	P-Value
**Age (y)**	37.04 ± 13.90	36.96 ± 13.23	36.97 ± 12.92	0.91
**Sex (male/female)**	35/35	34/36	33/37	0.87
**Height (cm)**	168.11 ± 8.70	168.49 ± 8.64	168.76 ± 8.48	0.38
**Weight (Kg)**	67.39 ± 12.28	68.09 ± 12.12	68.24 ± 12.53	0.27
**BMI (kg/m** ^**2**^ **)**	23.77 ± 3.21	23.70 ± 3.12	23.95 ± 3.10	0.147

^a^ Values are expressed as mean ± standard deviation.

Six people (8.6%) in group K, 15 people (21.4%) in group L, and 28 people (40%) in group N experienced a cough, and there was a significant difference between the three groups in terms of having a cough (P = 0.001) (Table 2). ANOVA with post-hoc Bonferroni correction was performed for between-group P-value comparison: Group K and group L (P = 0.03), group K and group N (P = < 0.001), group L and group N (P = 0.017). The incidence of FIC was 23.3% in this study.

**Table 2.** Incidence of Cough

Variables	K Group (70)	L Group (70)	N Group (70)	P-Value
**Incidence of cough; n (%)**	6 (8.6)	15 (21.4)	28 (40)	0.001

In terms of the severity of the cough, 3 patients (4.3%) in group L and 9 patients (12.9%) in group N experienced the severe form of cough, and there was a significant difference between the groups in this regard (P = 0.001) (Table 3). ANOVA with post-hoc Bonferroni correction was performed for between-group P-value comparison: Group K and group L (P = 0.123), group K and group N (P = < 0.001), group L and group N (P = 0.09).

**Table 3.** Severity of Cough

Variables	K Group (70)	L Group (70)	N Group (70)	P-Value
**Severity of cough; n (%)**				0.001
Mild	4 (5.7)	7 (10)	12 (17.1)	
Moderate	2 (2.9)	5 (7.1)	7 (10)	
Severe	0	3 (4.3)	9 (12.9)	

The average cough onset time in group K was 17.5 seconds, in group L was 16.7 seconds, and in group N was 13.1 seconds. There was a significant difference between the K and N groups in terms of the onset time of cough (P = 0.042). In general, the average cough onset time in the study subjects was 7.47 ± 3.37 (0 - 40) seconds. In terms of HR and arterial SpO_2_ at different times, there was no significant difference between the three study groups (P > 0.05). However, arterial BP at T1 (3 minutes after fentanyl injection) and T2 (1 minute after intubation) showed a significant difference between the three groups (P = 0.003, 0.001) (Table 4). Additionally, no unpleasant events such as SpO_2_ drop, aspiration, cyanosis, apnea, or severe changes in BP and HR during coughing occurred in any of the three groups.

**Table 4.** Vital Signs ^a^

Variables	K Group (70)	L Group (70)	N Group (70)	P-Value
**Heart rate**				
Minute 0 (before drug injection)	88.51 ± 13.72	84.59 ± 15.24	87.49 ± 14.29	0.25
Three minutes after fentanyl injection	74.19 ± 11.98	74.24 ± 14.73	78.91 ± 14.86	0.08
One minute after intubation	77.21 ± 15	79.91 ± 16.38	79.93 ± 14.55	0.485
**Mean arterial blood pressure**				
Minute 0 (before drug injection)	99.14 ± 15.1	101.76 ± 15.5	103.47 ± 14.16	0.123
Three minutes after fentanyl injection	90.81 ± 12.23	95.27 ± 12.69	98.17 ± 12.03	0.003
One minute after intubation	90.99 ± 12.23	96 ± 9.49	98.27 ± 12	0.001
**Arterial oxygen saturation**				
Minute 0 (before drug injection)	94.27 ± 4.28	94.53 ± 4.4	94.1 ± 4.2	0.84
Three minutes after fentanyl injection	96.1 ± 2.94	97.07 ± 2.44	96.27 ± 2.9	0.1
One minute after intubation	96.94 ± 3.1	96.01 ± 2.14	96.1 ± 3	0.54

^a^ Values expressed as mean ± standard deviation.

## 5. Discussion

In this study, the authors found that the incidence of FIC is 23.3%, and pre-injection of 0.5 mg/kg ketorolac can effectively reduce the occurrence and severity of cough induced by 3 mcg/kg intravenous fentanyl. There was a significant difference between the ketorolac and placebo groups in terms of the onset time of cough, but there was no significant difference between the three study groups in terms of HR and arterial SpO_2_ at different times. The average arterial BP was higher in group N (normal saline) than in group L, and higher in group L than in group K. In a study by Shuying et al., drugs such as lidocaine, ketamine, fentanyl priming dose, and propofol had a significant effect in preventing opioid-induced cough compared to the control group (20), which was supported by the present study. In terms of cough severity, 3 individuals (4.3%) in group L (lidocaine injection) and 9 individuals (12.9%) in group N (normal saline injection) experienced severe coughing, and there was a significant difference between the groups in this regard. Both intravenous ketorolac and lidocaine have a significant effect on reducing the severity and incidence of coughing, although ketorolac was more effective than lidocaine in reducing cough incidence.

In a study conducted by Cho et al., it was observed that the prevalence of coughing was higher in individuals using fentanyl compared to those receiving normal saline. Furthermore, the frequency of coughing was higher in the remifentanil group than in the alfentanil group, although there was no significant difference in cough severity between these two drug groups (6). These findings are consistent with our study, which also revealed a higher prevalence of coughing in the normal saline injection group (Table 3). It is important to note that the amount of coughing caused by different combinations of fentanyl may vary, underscoring the need to carefully consider this factor before administering these drugs to prevent more severe side effects, such as coughing.

In a study conducted by Ozmen et al., coughing was observed in 8 patients in the 2-cc normal saline group, 3 patients in the 1 mg/kg lidocaine group, and 1 patient in the 45.5 mg pheniramine maleate group. The study concluded that pheniramine maleate was more effective than placebo in reducing coughing caused by fentanyl, similar to lidocaine (30). This finding is consistent with our study, which also observed a higher frequency of coughing in the normal saline group compared to the lidocaine group. The study emphasizes that appropriate pain management in combination with anesthesia drugs can have significant effects in reducing their side effects, including coughing. Intravenous administration of lidocaine has been shown to reduce reflex coughing as well.

In another study by Guler et al., three groups were assigned to receive lidocaine 1 mg/kg, ketamine 0.5 mg/kg, or placebo one minute before the injection of fentanyl. The results showed that compared to the placebo group, the intensity of coughing was significantly reduced in the lidocaine and ketamine groups after injecting 1.5 mcg/kg of fentanyl over 2 seconds (4). Our study's results also suggest that administering lidocaine alongside fentanyl can reduce the intensity of coughing caused by fentanyl. Lidocaine is commonly used to suppress the cough reflex in procedures such as intubation and bronchoscopy by inhibiting cough receptors in the airway pathways without affecting hemodynamics.

Tian et al. studied the effect of pretreatment with ketorolac on cough induced by sufentanil. The intervention group received 0.5 mg/kg ketorolac 5 minutes before anesthesia induction, while the control group received normal saline. All patients received sufentanil, followed by propofol and vecuronium. One minute after the ketorolac injection, cough severity and frequency were lower in the intervention group (33). The use of painkillers alongside fentanyl not only reduces the incidence and severity of coughing but also maintains the hemodynamics and vital signs of patients. This is crucial to prevent complications, such as hemodynamic changes, during anesthesia and patient recovery.

In a study by Lin et al., it was found that pre-treatment with remifentanil or painkillers can safely and effectively reduce the incidence and severity of coughing caused by sufentanil during anesthesia induction (1). The use of an appropriate alternative method to prevent and reduce the severity of coughing caused by fentanyl is a crucial aim of these studies. The present study proposes the use of a combination of drugs, including painkillers, alongside fentanyl to achieve this aim.

In further investigations in our study regarding the level of coughing in different study groups, the mean cough onset time in group K was calculated as 17.5 seconds, in group L as 16.7 seconds, and in group N as 13.1 seconds. There was a significant difference in cough onset time between the K and N groups, which was not measured in other studies. According to Du et al.'s study, an initial fentanyl dose of 0.5 mcg/kg effectively reduces the incidence and severity of cough, but doses of 1 or 1.5 mcg/kg have no effect on FIC (7). Similarly, Jong In Han's study found that cough severity was significantly lower in patients receiving a lower dose of fentanyl (1 mcg/kg) compared to those receiving a higher dose (2 mcg/kg), but there was no significant difference in intubation conditions between the two groups (14). Although our study did not investigate the effect of fentanyl dose, these findings highlight the need to address the adverse effects of this drug and find ways to reduce or prevent coughing caused by fentanyl.

In our study, we found that the incidence of cough caused by fentanyl did not significantly vary by gender and age. Another study by Oshima et al. investigated the association between demographic characteristics, smoking history, bronchial asthma or obstructive pulmonary disease, use of ACE inhibitors, anesthesia method including premedication with anti-anxiety drugs, previous epidural lidocaine and atropine administration, priming dose of vecuronium, and intravenous fentanyl dose with cough caused by fentanyl. The results of this study showed that cough caused by fentanyl was independently associated with aging, smoking history, and previous epidural lidocaine injection, but not with gender and other variables. This study's findings suggest that cough caused by fentanyl may be affected by various risk factors that patients may have during surgery and the use of anesthesia drugs. Both our study and Dr. Oshima's study showed that the use of lidocaine with fentanyl anesthesia reduced coughing side effects and created hemodynamic stability, particularly in individuals with previous risk factors, to prevent them from being affected by adverse effects (24).

This study found no significant difference in HR and arterial SpO_2_ among the three study groups at different time intervals. However, there was a significant difference in arterial BP 3 minutes after fentanyl injection and 1 minute after intubation between the three groups, with the mean being higher in the normal saline group than in the lidocaine injection group, and the lidocaine injection group higher than the ketorolac injection group. Another study by Gillen Guler showed no significant difference in BP, HR, and SpO_2_ between groups that received lidocaine, ketamine, or a placebo before fentanyl injection (4). Our study suggests that the increase in arterial BP may be due to coughing caused by fentanyl, and the use of a combination of anesthetic drugs with painkillers can prevent this side effect to some extent by reducing the intensity and occurrence of FIC ing. However, further research is needed to investigate this issue, given the conflicting results of the two studies.

Our study demonstrated that no adverse events occurred in any of the three study groups during coughing, which is similar to the findings of Min-qing Liu's study, where there was no significant difference in side effects between groups (10). Therefore, it is essential to use methods that minimize the side effects of fentanyl, such as increased abdominal and intracranial pressure, pneumothorax, and postoperative nausea and vomiting. Additionally, the speed of drug injection may also play a role in causing cough, which was not addressed in our study. Another study by Sun et al. found that prophylactic intravenous lidocaine administration can reduce the incidence and severity of cough caused by narcotics without causing any undesired side effects (23). This study is consistent with our results, which showed that lidocaine reduced the severity of cough caused by fentanyl and maintained stable hemodynamics. Therefore, using drugs like lidocaine alongside fentanyl can be effective in preventing adverse effects of anesthesia drugs.

This study was conducted at a single center, which may limit the generalizability of the findings. However, this design allowed for the exclusion of inter-observer variability, as all evaluations were performed by a single observer.

### 5.1. Conclusions

Accordingly, in this study, we concluded that the administration of ketorolac and lidocaine reduces the incidence and severity of coughing caused by fentanyl. In particular, no cases of severe coughing were observed in individuals who received ketorolac, and therefore, ketorolac can be used to prevent coughing as a side effect of fentanyl, especially when lidocaine use is contraindicated. Additionally, the absence of other adverse events such as oxygen desaturation, aspiration, cyanosis, apnea, and significant changes in BP and HR during coughing was emphasized in all three groups, indicating the significant impact of these drugs.

## Data Availability

The data are available upon request from the corresponding author during submission or after publication.

## Clinical Trial Registration Code:

The study was registered in Iranian Registry of Clinical Trial with registry number IRCT20220102053599N1.
